# Antidepressant Treatment-Induced State-Dependent Reconfiguration of Emotion Regulation Networks in Major Depressive Disorder

**DOI:** 10.3389/fpsyt.2021.771147

**Published:** 2022-01-06

**Authors:** Lei Zhao, Donglin Wang, Shao-Wei Xue, Zhonglin Tan, Hong Luo, Yan Wang, Hanxiaoran Li, Chenyuan Pan, Sufen Fu, Xiwen Hu, Zhihui Lan, Yang Xiao, Changxiao Kuai

**Affiliations:** ^1^Centre for Cognition and Brain Disorders, The Affiliated Hospital of Hangzhou Normal University, Hangzhou, China; ^2^Institute of Psychological Science, Hangzhou Normal University, Hangzhou, China; ^3^Zhejiang Key Laboratory for Research in Assessment of Cognitive Impairments, Hangzhou, China; ^4^Affiliated Mental Health Center & Hangzhou Seventh People's Hospital, Zhejiang University School of Medicine, Hangzhou, China

**Keywords:** major depressive disorder, recurring functional interaction patterns, emotion regulation, antidepressants, dynamic functional connectivity

## Abstract

Deficits in emotion regulation are the main clinical features, common risk factors, and treatment-related targets for major depressive disorder (MDD). The neural bases of emotion regulation are moving beyond specific functions and emphasizing instead the integrative functions of spatially distributed brain areas that work together as large-scale brain networks, but it is still unclear whether the dynamic interactions among these emotion networks would be the target of clinical intervention for MDD. Data were collected from 70 MDD patients and 43 sex- and age-matched healthy controls. The dynamic functional connectivity (dFC) between emotion regions was estimated via a sliding-window method based on resting-state functional magnetic resonance imaging (R-fMRI). A k-means clustering method was applied to classify all time windows across all participants into several dFC states reflecting recurring functional interaction patterns among emotion regions over time. The results showed that four dFC states were identified in the emotion networks. Their alterations of state-related occurrence proportion were found in MDD and subsequently normalized following 12-week antidepressant treatment. Baseline strong dFC could predict the reduction rate of Hamilton Depression Rating Scale (HAMD) scores. These findings highlighted the state-dependent reconfiguration of emotion regulation networks in MDD patients owing to antidepressant treatment.

## Introduction

Major depressive disorder (MDD) is characterized by persistent low mood and loss of interest (1). As a disorder that involves extensive affective aberrations, MDD must be elucidated by its brain mechanisms of emotion dysregulation (2). Human health and well-being benefit from appropriate emotion regulation that allows one to adaptively control the intensities, durations, and types of emotional experiences and behavioral responses evoked by external or internal stimuli (3, 4). Therefore, emotion regulation in healthy individuals and dysregulation in MDD have very important research significance (5). Previous studies have been trying to uncover how the brain generates and manages emotion, especially which depends largely on the knowledge about its large-scale organization (6). Functional magnetic resonance imaging (fMRI) has become a dominant tool for understanding emotion regulation and some specific brain loci that engage in the generation and regulation of emotion have been revealed (7). The fact that individual brain regions usually contribute to more than one specific emotion and that one emotional task can activate multiple brain regions simultaneously point out the promise of network-level representation for emotion (8). The characterization of large-scale brain networks (LBNs) and their functional interactions is becoming an increasingly common gateway to uncover how emotions are organized in the brain (9). Applying a meta-analytic k-means clustering approach on 385 experiments from 107 published papers, a recent study identified four LBNs based on convergent brain activation patterns during emotion regulation (10). Each LBN represents an activation pattern that occurs in multiple emotion regulation experiments. These four data-driven LBNs underwent also functional decoding to validate their differences in functional characterization. Especially, the first network mainly consisted of the lateral dorsal prefrontal cortex, which was associated with working memory, explicit memory, reasoning, and inhibition. The second network exhibited convergent activation in a lateralized ventral prefrontal network, which was primarily linked to language processes. The third network was based on convergent activation in the insula, precuneus, and posterior cingulate cortex, which was associated with the action, perception, and interoception domain. The last network consisted of subcortical regions such as the bilateral amygdala, left parahippocampus, and bilateral fusiform gyrus. This network indicated a focus on the emotion and memory domains. This progress provided relatively complete emotion regulation networks to enable further research on emotion dysfunction in MDD based on the functional interactions among hierarchical neural systems.

Despite the amount of effort to develop antidepressant treatments, treatment effectiveness has not been increased sufficiently in recent decades (11). This is partly because of the obscure knowledge with respect to the neural bases of MDD and its effective treatment (12). Emotion regulation is a key predictor for the course of MDD (13), and the strategy required to regulate the emotion of MDD patients varies with their severity of depressive symptoms (14). Adaptive emotion regulation could enhance the therapeutic effect on MDD and hence enable itself to be a promising target in clinical intervention for MDD (15, 16). Furthermore, numerous previous studies using resting-state functional magnetic resonance imaging (R-fMRI) have supplied clues about the association between the therapeutic outcomes of antidepressants and areas known to be involved in emotion generation or regulation. For example, effective antidepressant treatment for MDD patients was relevant with lower functional connectivity (FC) between the subcallosal cingulate cortex and ventromedial prefrontal cortex (17). Hyperconnectivity of the amygdala in MDD patients was reduced following 8-week antidepressant treatment (18). The FC between the medial prefrontal cortex and posterior cingulate cortex at baseline was predictive of the remission status of antidepressant treatment (19). However, most of these studies relied on the assumption of temporal stationarity during the entire R-fMRI scan. Time-averaged or static functional connectivity (sFC) may limit the detection of time-varying functional interaction patterns.

Emotion regulation itself conveys the adaptive nature of emotion from one aspect that demands one to reorganize his/her mind or behavior to deal with the changing environment for the purpose of achieving and maintaining well-being (20, 21), which obviously needs to be instantiated by different functional interaction patterns among LBNs. Emotion dysfunction usually occurs when one's emotion regulation dynamics fail to achieve short- or long-term goals (4). Therefore, investigating the mental illness labeled emotion dysfunction from the view of emotion regulation dynamics will deepen our comprehension of MDD pathology. Characterizing the dynamic functional interactions among LBNs that represent emotion regulation may help to elucidate how emotions are controlled or become out of control in the course of the emotion process. A dynamic functional connectivity (dFC) analysis approach has been proposed to capture the course of dynamic interactions among LBNs (22). After characterizing several recurring functional interaction patterns over R-fMRI scan time, high-level summaries would be obtained based on the temporal configurations of them (23), which were thought to load important physiological significance (24, 25). The human brain organizes and integrates various neural systems across multiple spatiotemporal scales constantly to achieve personal adaptability to internal or external environments (26). It makes this method significant when examining the neural mechanisms underlying kinds of disorders including schizophrenia (27), MDD (28), chronic pain (29), and attention deficit hyperactivity disorder (30). However, it is still unclear whether this method could be utilized to delineate the dynamic interactions among the LBNs serving for emotion regulation, and more importantly, whether the temporal configuration of the recurring functional interaction patterns would be a target of clinical intervention for MDD.

In the present study, we explored the recurring functional interaction patterns among four LBNs enrolled in emotion regulation. We hypothesized that there would be several recurring functional interaction patterns over time during the R-fMRI scans and that MDD would be associated with the abnormalities in such patterns. Furthermore, the abnormal characteristics of these patterns would be reconfigured in MDD patients by the administration of antidepressants. Additionally, we constructed multivariate linear regression models to investigate which patterns had the potential to predict the outcome of antidepressant treatment in MDD.

## Materials and Methods

### Participants

Seventy patients with MDD (49 females/21 males) were enrolled from the Department of Psychiatry of Hangzhou Seventh People's Hospital and the Department of Psychiatry at the Affiliated Hospital of Hangzhou Normal University. The course of interview and diagnosis for all MDD patients were accomplished by certified psychiatrists using the Mini-Neuropsychiatric International Interview (MINI) based on the Diagnostic and Statistical Manual of Mental Disorders (Fourth Edition) (DSM-IV) criteria. Forty-three healthy controls (HC, 27 women/16 men) matched in age and sex were enrolled from the local community. The 24-item Hamilton Depression Rating Scale (HAMD) was used to evaluate the depression severity of all participants. Individuals who had a neurological or medical illness, were pregnant or breastfeeding, showed severe suicidal tendencies, or displayed substance dependence were not included in the present study. Those who had excessive head motion were also excluded. All study procedures were performed in accordance with the Declaration of Helsinki on Ethical Principles and approved by the local Institutional Review Boards (IRB No.20150729) of Hangzhou Normal University. All subjects provided written informed IRB-approved consent before participating in study procedures.

### Treatment Outcomes

After medical and psychiatric assessments, participants who met the inclusion criteria completed both magnetic resonance imaging (MRI) scans at baseline. MDD patients then began to receive antidepressant treatment with typical selective serotonin reuptake inhibitors (SSRIs). The medication doses were prescribed and adjusted by the treating clinicians according to routine clinical practice and followed the recommended dose ranges. During 12-week antidepressant treatment, 19 patients were administered with atypical antipsychotics and benzodiazepines to improve quality of life. After 12 weeks of antidepressant treatment, 37 MDD patients underwent a repeated MRI scan and clinical assessment. These MDD patients were divided into responsive depression group (RDG) and non-responsive depression group (N-RDG) according to whether the reduction rate of the HAMD scores at the end of 12 weeks was >50% or not. HC did not undergo a repeated MRI scan after 3 months.

### Image Acquisition and Preprocessing

All MRI data were collected using a 3.0T GE scanner (General Electric, Waukesha, WI, USA) at the Center for Cognition and Brain Disorders of Hangzhou Normal University. The participants were asked to stay relaxed with their eyes closed, and not to fall asleep in particular, and their heads were fixed using a tight cushion. Functional images were obtained in an interleaved order using a T2^*^-weighted gradient-echo echo-planar-imaging (EPI) sequence (TR/TE = 2,000/22, flip angle = 77°, field of view = 240 × 240 mm^2^, matrix = 96 × 96, 2.5 mm isotropic spatial resolution with 42 slices and 240 volumes). A high-resolution T1-weighted anatomical image in sagittal orientation using Fast Spoiled Gradient echo sequence (3D FSPGR, TR/TE = 9/3.66, flip angle = 13°, field of view = 240 × 240 mm^2^, matrix = 300 × 300, 0.8 mm isotropic voxels, 176 slices without interslice gap) was obtained for visualization and localization of the functional data.

The MRI data were processed using the DPARSF toolbox (http://www.rfmri.org/), SPM (http://www.fil.ion.ucl.ac.uk/spm/), and custom code written in MATLAB. The first 10 functional volumes were discarded to allow for signal equilibration and environmental adaptation. The remaining images were corrected for the time shifts among different acquisitions within each volume by sink interpolating volume slices. The participants who had a maximum displacement of higher than 2.5 mm in the x-, y-, or z-axes and an angular motion of higher than 2.5° during the entire scan were excluded from the study (31, 32). The mean framewise displacement (FD) for each participant was recorded and participants with mean FD exceeding 0.5 mm were excluded (33). The corrected images were then spatially normalized into the standard stereotactic space of the Montreal Neurological Institute (MNI) with a resampled voxel size of 3 × 3 × 3 mm^3^. Nuisance covariates including the white matter and cerebrospinal fluid (CSF) signal and Friston-24 motion parameters were regressed out from the time course of each voxel. Then, the images were smoothed with a 6-mm full-width at a half-maximum Gaussian kernel. The linear trends were removed by detrending the signals of each voxel. Temporal bandpass filtering was performed at a frequency range of 0.01–0.10 Hz. Finally, scrubbing was performed to reduce the noise derived from head motion (33). The signal at the “bad” time points was interpolated using a cubic spline with an FD threshold of 0.5 mm (34).

### Sliding-Window dFC Analysis

We performed dFC analysis for four brain networks or LBNs enrolled in emotion regulation that was revealed by a previous study (10). The four LBNs included 36 brain areas with distinct spatial distributions and functional profiles. As shown in Table 1, their spherical regions of interest (ROIs) with a 5 mm radius were obtained based on the MNI coordinates of the peak voxels in the corresponding areas and used for the following analysis.

**Table 1:** The MNI coordinates of the ROIs within four large-scale brain networks enrolled in emotion regulation.

**ID**	**Regions of interest**	**X**	**Y**	**Z**
	**LBN 1**			
1	Superior Frontal Gyrus (L)	0	24	50
2	Middle Frontal Gyrus (R)	40	24	42
3	Inferior Parietal Lobule (R)	58	−52	38
4	Inferior Parietal Lobule (L)	−58	−50	44
5	Middle Frontal Gyrus (L)	−36	52	−2
6	Middle Frontal Gyrus (L)	−42	14	48
7	Middle Frontal Gyrus (R)	42	46	−8
8	Insula (R)	36	16	6
9	Cingulate Gyrus (R)	2	−22	30
10	Precuneus (R)	10	−64	36
	**LBN 2**			
11	Inferior Frontal Gyrus (L)	−46	24	−8
12	Superior Frontal Gyrus (L)	−4	10	62
13	Inferior Frontal Gyrus (R)	50	28	−8
14	Superior Temporal Gyrus (L)	−46	−52	28
15	Middle Temporal Gyrus (L)	−54	−34	−2
16	Middle Frontal Gyrus (L)	−44	6	50
17	Superior Frontal Gyrus (L)	−30	48	26
18	Caudate (L)	−16	10	12
19	Tuber (R)	36	−60	−30
	**LBN 3**			
20	Amygdala (L)	−22	−4	−16
21	Amygdala (R)	24	−4	−18
22	Fusiform Gyrus (R)	40	−46	−18
23	Thalamus (R)	6	−26	0
24	Fusiform Gyrus (L)	−38	−54	−14
25	Parahippocampal Gyrus (L)	−22	−28	−4
26	Medial Frontal Gyrus (B)	0	54	−10
27	Inferior Occipital Gyrus (L)	−42	−76	−6
	**LBN 4**			
28	Postcentral Gyrus (L)	−58	−22	32
29	Insula (L)	−44	−4	10
30	Superior Parietal Lobule (L)	−28	−52	56
31	Postcentral Gyrus (R)	62	−22	30
32	Cuneus (L)	−10	−76	22
33	Middle Occipital Gyrus (L)	−48	−74	2
34	Thalamus (R)	10	−26	−4
35	Precuneus (R)	28	−60	38
36	Posterior Cingulate (R)	16	−56	16

*B, bilateral; L, left; R, right; LBN, large-scale brain network*.

**Figure 1:**
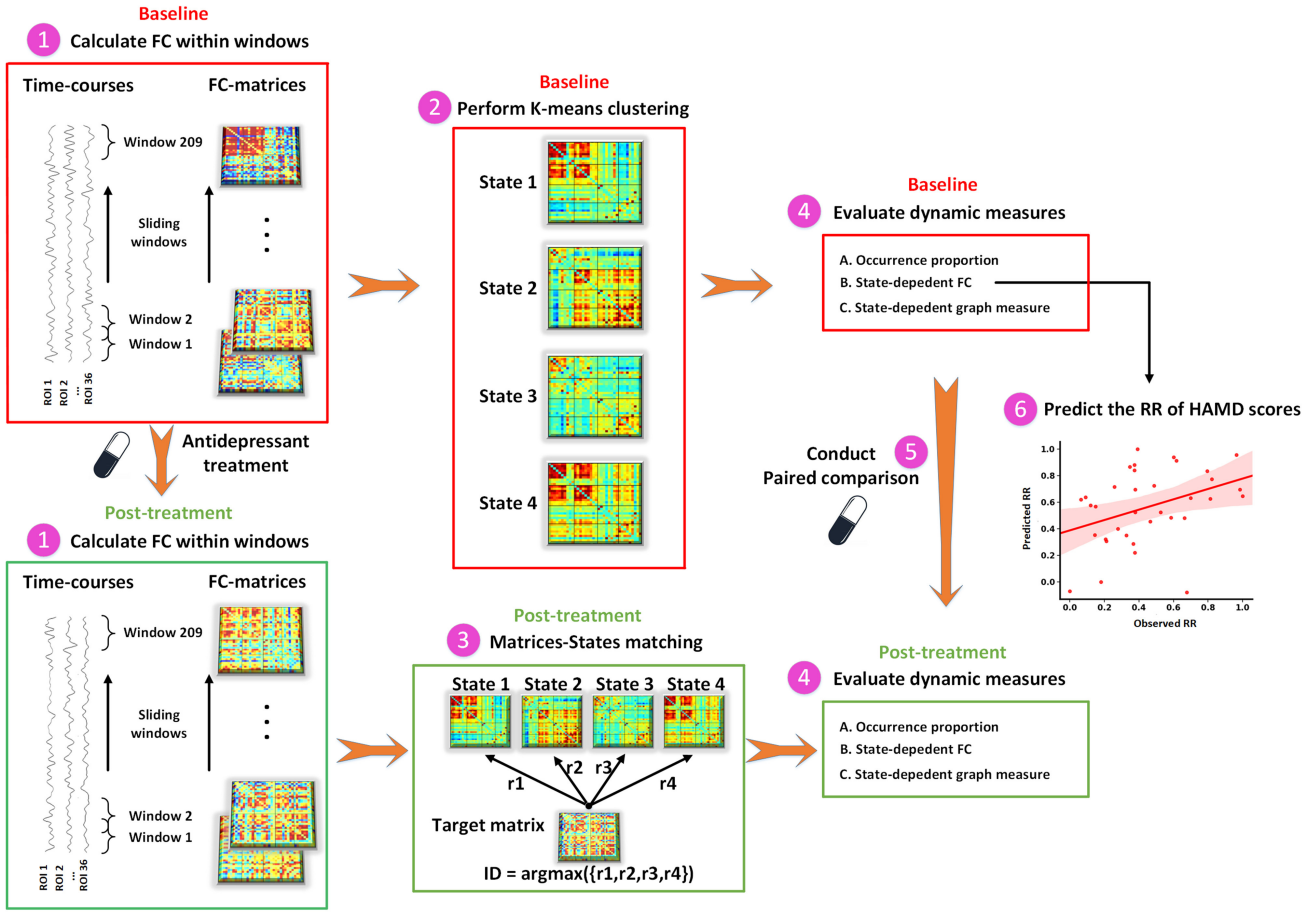
The schematic diagram of dFC analysis. dFC, dynamic functional connectivity; FC, functional connectivity; HAMD, Hamilton Depression Rating Scale; ROI, region of interest; RR, reduction rate.

As shown in Figure 1, the dFC was measured via the sliding-window approach. The window size was set to 22 TRs (44 s) and data in each window were convolved with a Gaussian (σ = 3 TRs) (35). For each subject, 209 consecutive windows were obtained by setting the shift step as 1 TR (2 s). The covariance matrix was estimated using the graphical LASSO (Least absolute shrinkage and selection operator) method on the windowed data (36). For each subject, the L1-regularization penalty was set using five-fold cross-validation with 50 repetitions. Finally, FC matrices were Fisher z-transformed to improve statistical normality.

To capture the recurring functional interaction patterns characterized as connectivity matrix over time, we applied a k-means clustering method based on dFC values to classify all windows across all participants into several distinct states. The correlation distance function was used to measure the distances between each window and cluster centroids. The optimal number of states was determined by using the elbow criterion ranging from 2 to 9. To avoid locally optimal solutions, the clustering procedure was repeated 100 times. At baseline, the between-regional interaction pattern at each state was obtained by averaging connectivity matrices of all windows within that state. In an iterative process, one individual's connectivity matrix was selected from all time windows at the post-treatment and compared against the connectivity matrix of each state at baseline to find the matrix that was maximally similar, and then the window at the post-treatment was assigned to the state with their maximum correlation coefficients.

Dynamic indices were assessed for each participant, including (1) occurrence proportion, measured as the proportion of time window number in a particular state to the total number of time windows during the scan (209 windows in the present study); (2) state-dependent alterations in FC; and (3) state-dependent alterations in graph-theory measure. We calculated the FC in each state separately and named it dFC strength. For each subject, the dFC strength in a state was acquired by averaging all the windows assigned to that state. Mean local efficiency was investigated to demonstrate the efficiency of information transfer in each functional interaction pattern. The local efficiency of each node was calculated using the GRETNA software (http://www.nitrc.org/projects/gretna) with the non-negative value of the weighted FC matrix as input (37). The sFC was also calculated to test whether state-dependent alterations could be detected in static functional interaction patterns.

### Predicting the Reduction Rate of HAMD Scores

In the present study, we constructed multivariate linear regression models to determine whether and which recurring functional interaction pattern could predict the reduction rate (RR) of HAMD scores. According to previous studies (31, 38), we employed a leave-one-out cross-validation (LOOCV) strategy to evaluate the performance of the regression models. For each iteration of LOOCV, the state-dependent FC of an MDD patient at baseline was applied to predict his/her RR of HAMD scores based on the regression model trained by remaining patients. Finally, Pearson's correlation coefficient between the observed and predicted RR of HAMD scores was calculated to evaluate the performance of the predictive model. An additional multivariate linear regression model was also constructed to examine the predictive ability of static functional interaction patterns for the treatment effect of antidepressants.

### Statistical Analysis

Statistical comparisons between MDD and HC at baseline were performed on dynamic measures via the general linear model (GLM), with age, sex, and mean FD as covariates. The paired-sample *t*-test was utilized to evaluate the longitudinal alterations of depression severity and abnormal dynamic measures in RDG and N-RDG following antidepressant treatment. The threshold of statistical significance was set at 0.05. The correction for multiple comparisons was performed using the false discovery rate (*p* < 0.05, FDR corrected). The one-sample *t*-test was utilized to examine the main FC of each state in the MDD and HC groups, respectively. The one-sample *t*-test results of the two groups were combined as a conjunction mask for subsequent between-group comparisons.

The statistical significance of the prediction results was assessed using permutation tests (39). Firstly, the observed RR of HAMD scores were randomly permuted beforehand across participants. Then, the aforementioned prediction processes were performed on the permuted dataset. This procedure was repeated 10,000 times. The performance of a regression model was assumed to be reliable if the result obtained by the regression model trained on the true dataset was higher than the 95% confidence interval of the regression model trained on a randomly permuted dataset.

## Results

### Demographic and Clinical Data

The demographic and clinical characteristics of the participants were summarized in Table 2. MDD patients were matched with HC on gender (*t* = 0.629, *p* = 0.428), age (*t* = −1.217, *p* = 0.226), and mean FD (*t* = −1.041, *p* = 0.300). Following 12-week antidepressant treatment, a total of 37 of 70 MDD patients completed a repeated MRI scan and HAMD assessment. A significant reduction on depression severity was found (paired *t*-tests, *t* = −9.479, *p* < 0.001). Twenty-three (62.16%) MDD patients achieved the responsive criteria and were then grouped into RDG. The remaining 14 sMDD (37.84%) patients were placed into N-RDG.

**Table 2:** Demographic and clinical data.

**Characteristics**	**MDD**	**HC**	***p*-value**	***t*/χ^2^ value**
Sex (male/female)	70 (21/49)	43 (16/27)	0.428^a^	0.629
Age (years)	26.93 ± 9.14	29.42 ± 12.55	0.226^b^	−1.217
Mean FD	0.11 ± 0.05	0.12 ± 0.06	0.300^b^	−1.041
HAMD	28.06 ± 6.67	1.35 ± 1.38	<0.001^b^	25.647
Duration of illness (months)	7.37 ± 12.60			
Episodes				
First	43			
Recurrence	27			
Medication history	29			

*The data were presented as the mean ± standard deviation. HC, healthy controls; MDD, major depressive disorder; HAMD, Hamilton Depression Rating Scale; FD, framewise-displacement*.

a *The p-value was obtained by a chi-square test*.

b *The p-value was obtained by a two-tailed two-sample t-test*.

### Between-Group and Follow-Up Comparisons of Dynamic Measures

**Figure 2:**
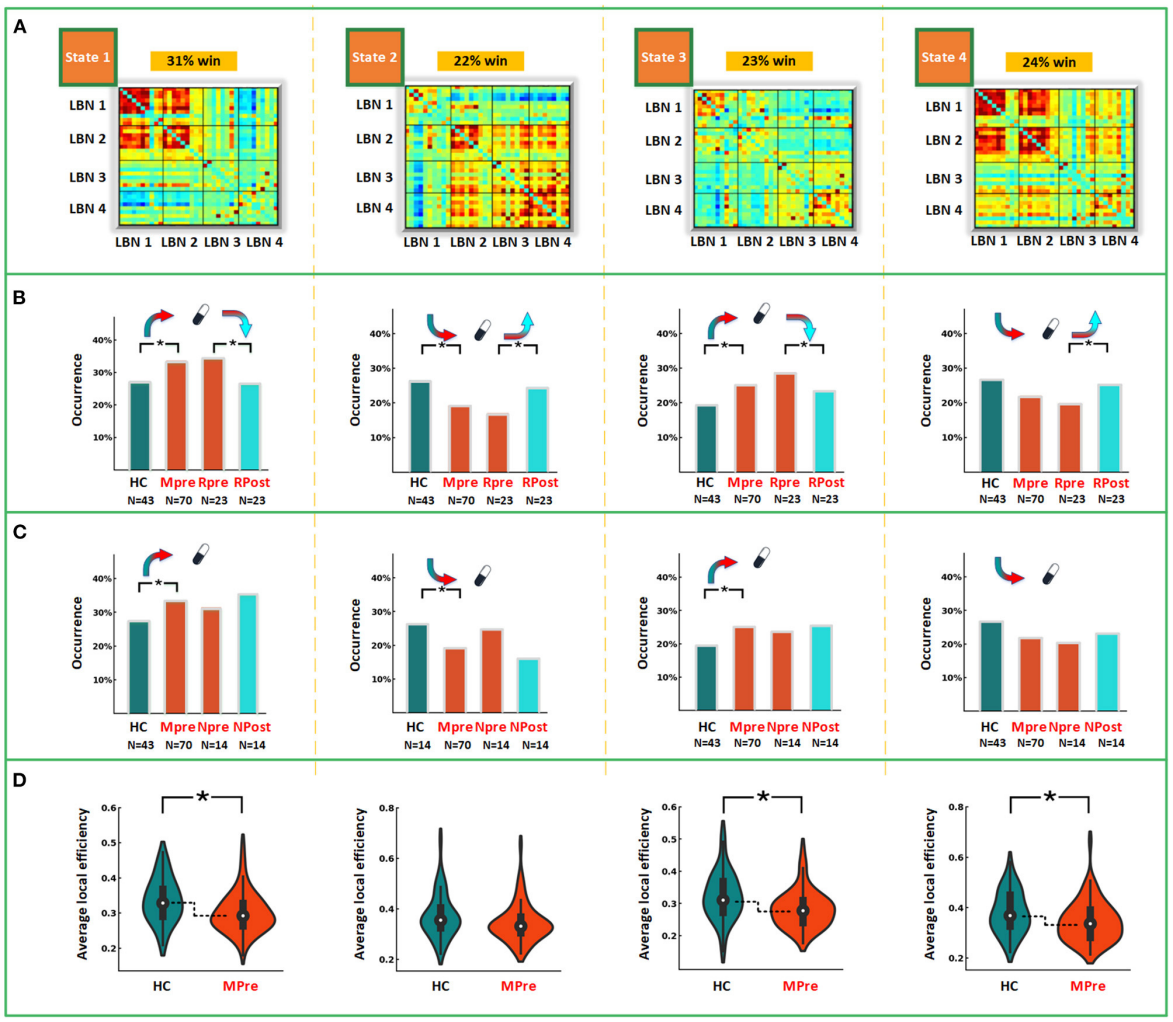
Intra- and between-group comparisons in dynamic measures. **(A)** Functional interaction patterns are represented by four states. The mean occurrence proportion of each state across all subjects was listed above each matrix. **(B)** The differences of occurrence proportion between MDD and HC, and between post-treatment and paired baseline responsive depression group. **(C)** The differences of occurrence proportion between MDD and HC, and between post-treatment and paired baseline non-responsive depression group. **(D)** The differences of average local efficiency between MDD and HC. LBN, large-scale brain network; win, window; HC, healthy controls; Mpre, major depressive disorder patients at baseline; Rpre, responsive depression group at baseline; Rpost, responsive depression group after treatment. Npre, non-responsive depression group at baseline; Npost, non-responsive depression group after treatment. ^*^p < 0.05 after FDR corrected.

**Figure 3:**
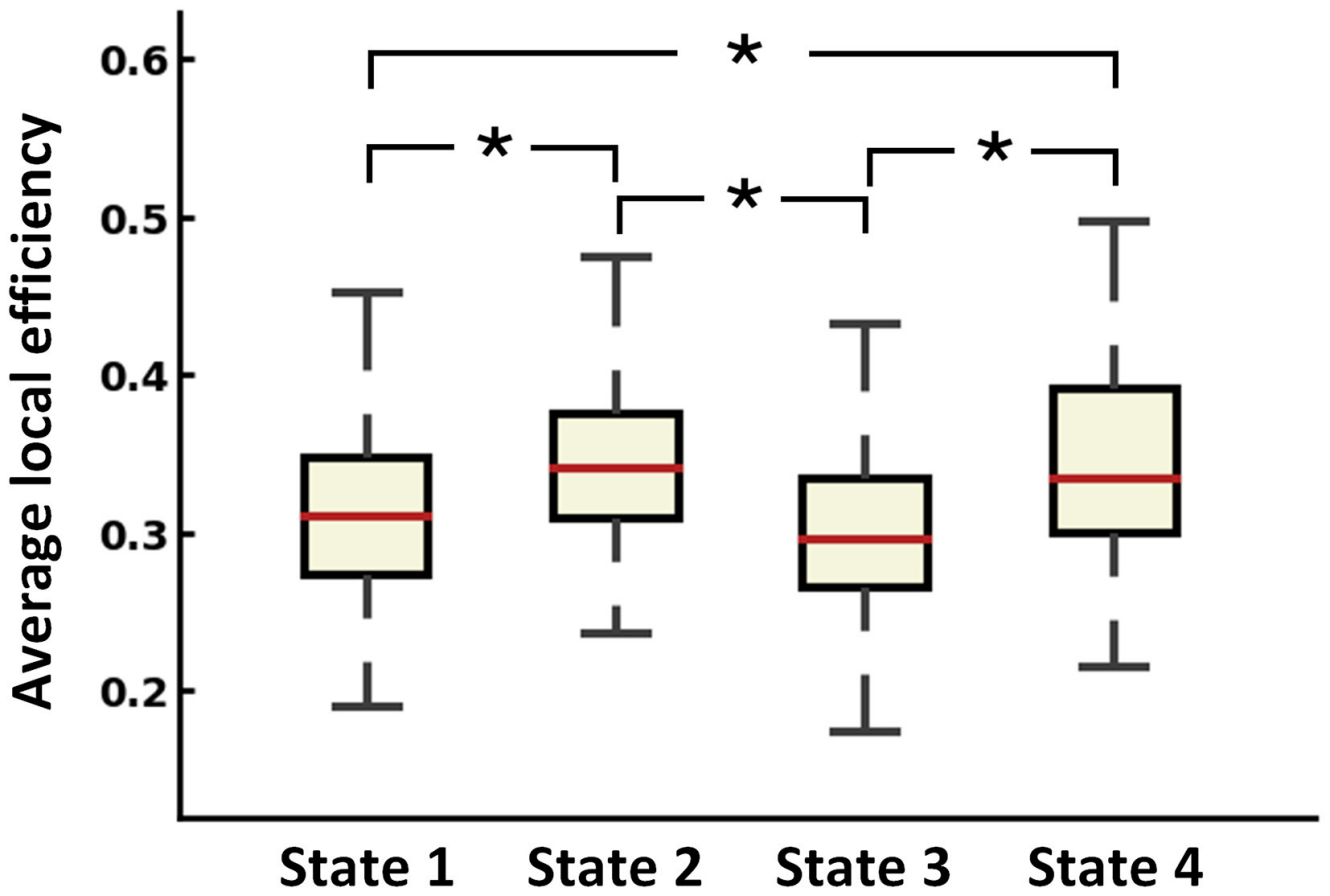
Average local efficiency of dFC states across all subjects. ^*^p < 0.05 after FDR corrected.

To identify the recurring inter-regional functional interaction patterns over R-fMRI scan time, all window slices were automatically divided into four distinct states by a k-means clustering method in the baseline phase. Figure 2A displays the recurring functional interaction patterns among four LBNs. State 1 exhibited strong FC between LBN 1 and LBN 2. In state 2, LBN 2, LBN 3, and LBN 4 were interconnected strongly. There was relatively sparse FC in state 3. Similar to state 1, state 4 had a strong FC between LBN 1 and LBN 2. Furthermore, stronger FC of LBN 4 with LBN 1 and LBN 2 was found in state 4 relative to state 1. Compared with state 1 and state 3, state 2 and state 4 exhibited significantly higher average local efficiency (*p* < 0.05, Figure 3).

**Figure 4:**
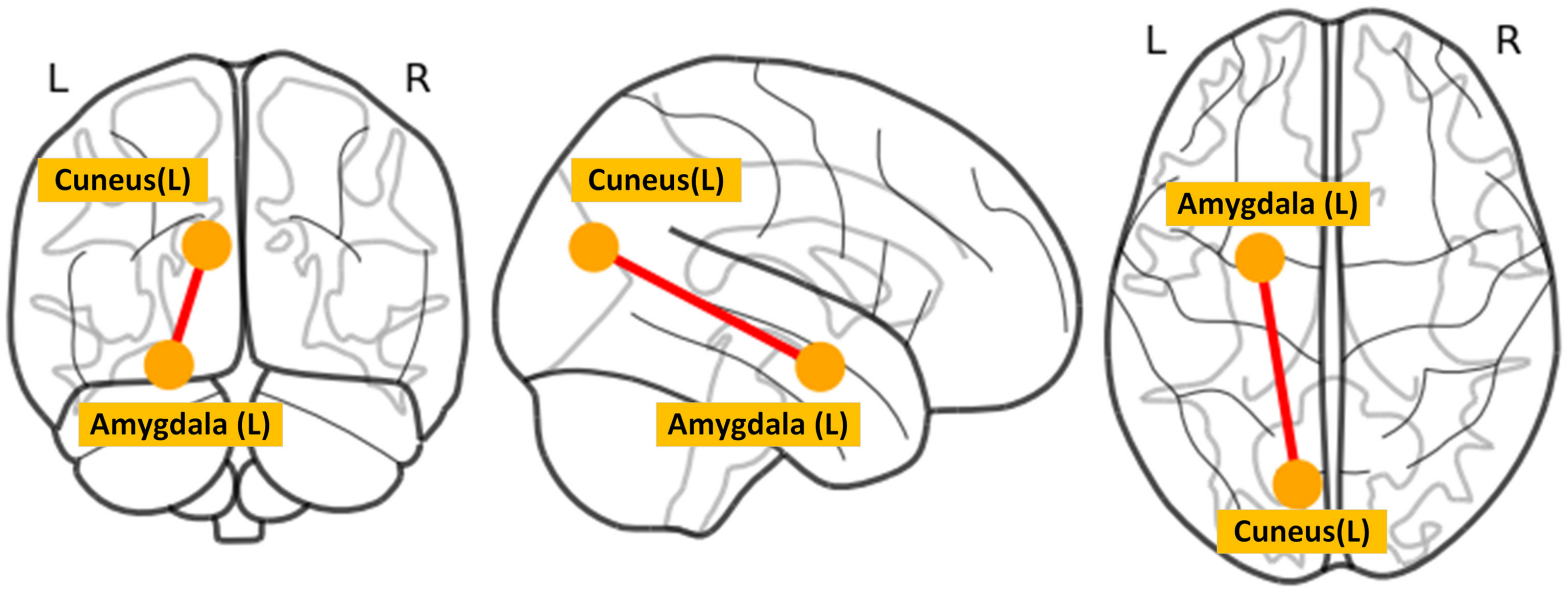
The between-group difference of dFC strength in state 2. L, left; R, right.

The between-group differences of the time to occurrence proportion of each state and average local network efficiency at baseline were shown in Figure 2. Compared with HC, MDD patients had a significantly more occurrence proportion in state 1 (*t* = 2.177, *p* = 0.032) and state 3 (*t* = 2.123, *p* = 0.036), but less occurrence proportion in state 2 (*t* = −2.627, *p* = 0.010). We observed that MDD patients showed significantly lower average local efficiency than HC in state 1 (*t* = −2.689, *p* = 0.008), state 3 (*t* = −2.969, *p* = 0.004) and state 4 (*t* = −2.313, *p* = 0.023). In state 2, MDD patients at baseline had significantly decreased dFC strength between the left amygdala and left cuneus (*t* = −4.070, *p* < 0.001) compared with HC (Figure 4). In sFC analysis, MDD patients at baseline did not show any significant alteration compared to HC (*p* > 0.05).

After 12 weeks of antidepressant treatment, MDD patients in RDG exhibited significant alterations in dynamic measures compared to those at baseline (Figure 2B). Specifically, patients in RDG showed increased occurrence proportion in state 2 (*t* = 2.505, *p* = 0.020) but decreased occurrence proportion in state 1 (*t* = −2.463, *p* = 0.022) and state 3 (*t* = −2.214, *p* = 0.037). No significant alteration was found in dFC strength and average local efficiency in each state (*p* > 0.05). Patients in N-RDG did not show significant alteration after treatment (*p* > 0.05).

### The Predicted RR of HAMD Scores

**Figure 5:**
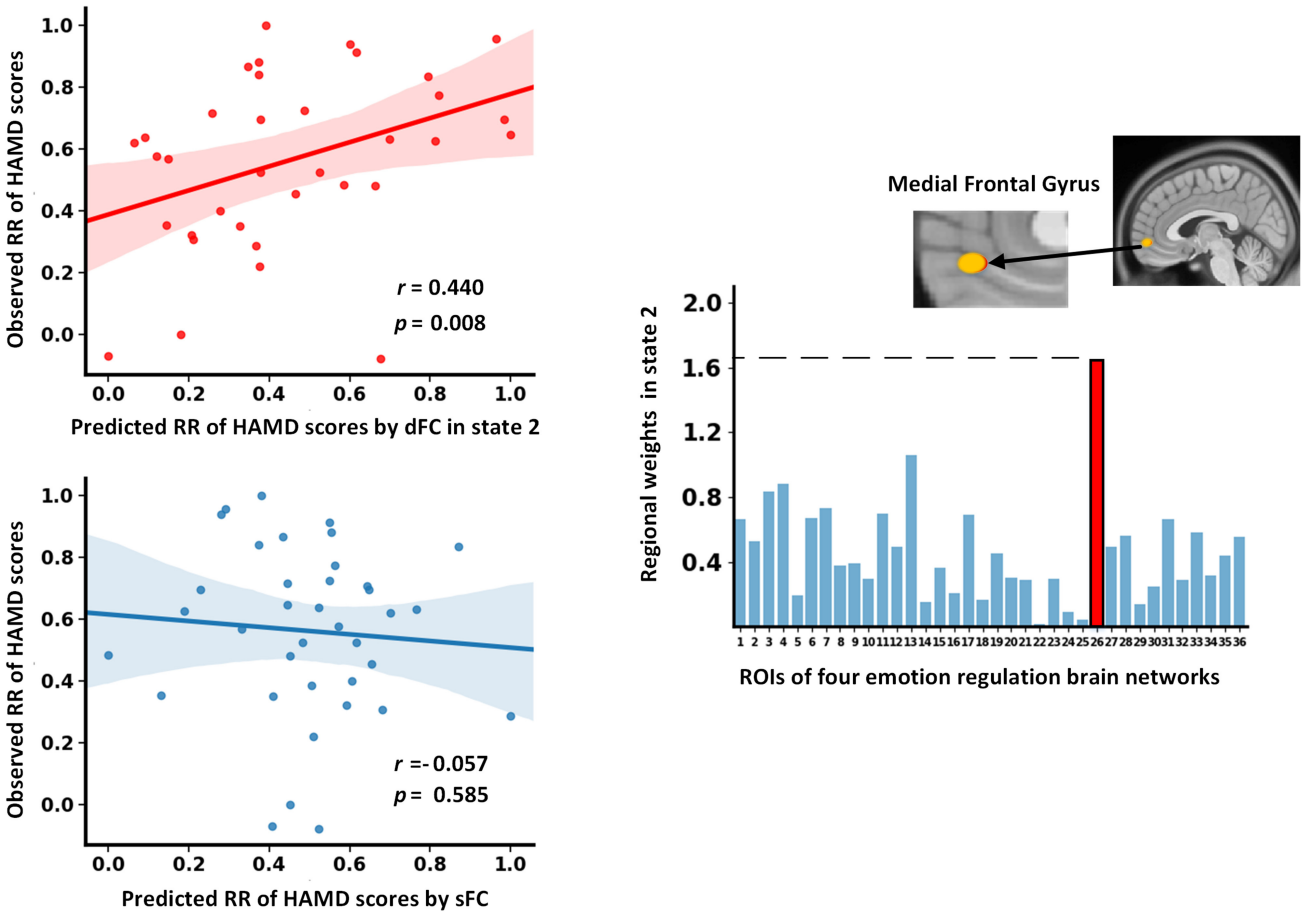
The results of dFC strength and sFC as features to predict the RR of HAMD scores. dFC, dynamic functional connectivity; HAMD, Hamilton Depression Rating Scale; RR, reduction rate; sFC, static functional connectivity.

As shown in Figure 5, the baseline dFC strength in state 2 of MDD patients significantly predicted their RR of HAMD scores following treatment (*r* = 0.440, *p* = 0.008). In the prediction model, the medial frontal gyrus (MFG) exhibited the highest prediction weight among all ROIs. In addition, the sFC did not predict the RR of HAMD scores significantly (*r* = −0.057, *p* = 0.585, Figure 5).

## Discussion

The present study investigated the recurring functional interaction patterns among four LBNs enrolled in emotion regulation. Four recurring functional interaction patterns over time were identified. Patients with MDD exhibited abnormal dynamic measures at baseline, including state occurrence proportion, state-dependent FC, and state-dependent average local efficiency. Administrated with antidepressants, the abnormal state occurrence proportion of patients in RDG was reconfigured toward a direction of healthy controls. The FC in state 2 at baseline predicted the RR of HAMD scores significantly wherein the connectivity of the MFG contributed the most to prediction.

### Recurring Functional Interaction Patterns

In the present study, four states represented distinct functional interaction patterns among four LBNs implicated in emotion regulation. State 1, which accounted for more than 30% of time windows, exhibited a strong interaction between LBN 1 and LBN 2. LBN 1, consisting mainly of the frontoparietal network including the dorsolateral prefrontal cortex (dlPFC), is implicated in working memory and response inhibition (10, 40). LBN 2 covers mainly the lateral ventral prefrontal network and is functionally associated with the cognition domain, especially language-related cognitive processing (10, 41). Approximately 90% of the experiments contributing to LBN 1 and LBN 2 were involved in the regulation phase of the emotion process (10). Most of these experiments implemented reappraisal as a regulation strategy. Though exerting strong interactions with each other, these two networks showed sparse connectivity with LBN 3 and LBN 4. Sparser connectivity could be found in state 3, of which relatively strong connectivity was concentrated between LBN 3 and LBN 4. The functional interaction patterns represented by state 1 and state 3 presented functional separation between the prefrontal cortex and subcortical regions.

State 2 had strong functional couplings between LBN 2, LBN 3, and LBN 4. As a network implicated in the cognitive regulation of emotion, LBN 1 did not show strong coordination with other LBNs. This could be interpreted by functional differences between the two regulatory networks along a dorsal (LBN 1)—ventral gradient (LBN 2) (42, 43). Previous studies have suggested that dorsal prefrontal regions play an important role in maintaining the goals of reappraisal in working memory during emotion regulation (42, 44) but are no longer required to maintain the monitoring of representations in working memory after finally selecting one from multiple representations of stimulus-appropriate reinterpretations which is mainly supported by ventral prefrontal regions (43, 45). Strong connectivity between the ventral and dorsal prefrontal regions is more likely to be found in the selection phase of goal-appropriate reappraisals where multiple reappraisals need to be represented in working memory (43). Therefore, the functional interaction pattern represented by state 2 might be related to the execution of cognitive reappraisals of emotion. LBN 3 consists of subcortical regions including the amygdala and is primarily involved in reactivity and generation of emotion. LBN 4 is composed of the areas relevant to emotion perception and interoception such as the insula, precuneus, and posterior cingulate cortex. It serves as a hub that plays an intermediary role in integrating information from the prefrontal cortex (LBN 1 and LBN 2) and subcortical regions (LBN 3). Functional synchronization between LBN 2 and LBN 3 might be associated with the reinterpretations for emotion reactivity, and LBN 4 maintained and coordinated information communication courses between them (46).

### Reconfiguration for Abnormal Dynamic Measures

MDD patients exhibited a lower occurrence proportion in state 2 but a higher occurrence proportion in state 1 and state 3. A lower occurrence proportion in state 2 seemly signified the difficulty of MDD patients to enter the functional interaction pattern in which the meaning of their emotional reactivity could be reinterpreted. By contrast, the functional separation between prefrontal and subcortical regions which appeared more frequently in temporal co-evolution of LBNs might be associated with less tendency or greater difficulties for MDD patients to regulate their emotions by using cognitive reappraisal. Cognitive reappraisal is an infrequently used adaptive emotion regulation strategy in MDD (2). Conversely, patients with MDD preferentially performed maladaptive emotion regulation strategies, for instance, rumination (47). Most of the regions in LBN 3 and LBN 4 are implicated in self-referential processing and have been reported to engage in rumination (48, 49). Superfluous immersions in relatively strong connectivity between these regions might be the neural basis for excessive rumination in MDD.

Decreased FC between the amygdala and cuneus was only found in state 2. The amygdala, a core region of the limbic system known to control emotion processing, plays a major role in the generation and regulation of emotion (50). Convergent evidence suggests that the amygdala is responsible for crucial functions related to depression including the processing of visual information elicited by emotional stimuli (51). The cuneus is located in the occipital lobe and is mainly involved in the processing of visual information (52). We speculated that decreased FC between the amygdala and cuneus in state 2 might reflect bias evaluation to emotion stimuli of MDD patients when performing cognitive reappraisal. We observed that four states had significantly different network efficiency. The functional interaction patterns represented by state 1 and state 3 presented lower efficiency of information transfer than state 2 and state 4. In addition, MDD patients had lower efficiency of information transfer in low-efficiency states (i.e., state 1 and state 3). Taken together, these state-dependent alterations indicated that the disruption of functional interaction patterns was not constant but intermittent.

After 12 weeks of antidepressant treatment, normalization for abnormal occurrence proportion was observed in MDD patients who responded to treatment. However, state-dependent alterations in FC and graph-theory measures were not modified by antidepressant treatment. This indicated that antidepressants might promote remission of MDD by modulating the dynamic interaction process between LBNs related to emotion regulation. Specifically, the prefrontal cortex displayed more frequent functional communication with subcortical networks compared to baseline in RDG. Such an effect was potentially relevant to more frequent employment of adaptive emotion regulation strategies (e.g., reappraisal) in MDD patients after antidepressant medication (53). However, reconfiguration for abnormal occurrence proportion was not found in those who did not respond to treatment. Similar to previous studies (54), our results, from the dynamic interaction process among LBNs, supported the view that adaptive emotion regulation is a key therapeutic target for effective treatment for MDD (55, 56).

### The Dynamic Interaction Pattern With Predictive Power

The static functional interaction pattern at baseline failed to predict the RR of HAMD scores after 12 weeks of treatment. Interestingly, the functional interaction pattern of state 2 at baseline significantly predicted the RR of HAMD scores in MDD patients. Although previous studies have underlined the classifying and predictive ability of dFC (57, 58), most of them focused on its temporal variation instead of recurring functional interaction patterns. Our findings highlighted the potential of state-based dFC analysis in developing biomarkers for clinical applications. In addition, the FC of the MFG contributed the most to the prediction. The MFG, a midline frontal region, is typically implicated in emotion regulation (42). It was thought to facilitate the generation of purposeful and adaptive behavior (59). Functional impairments in this area were reported and related to negatively biased attention in MDD patients (60). More importantly, a previous study demonstrated that structural alteration of the MFG was correlated with the improvement of depressive symptoms following cognitive-behavioral therapy (61). Our results provide further evidence that the MFG plays an important role in the effective treatment of depression.

### Limitations

Several limitations need to be considered in the present study. First, the sample size of MDD patients administrated with antidepressants was relatively small, which might reduce the generalizability of the work. Future studies with a larger sample size are imperative to assess the reproducibility of our findings. Second, the acquisition time of R-fMRI was 8 min. More time points might help to discover more subtle functional interaction patterns with a low occurrence proportion. Third, some patients withdrew from the study after baseline and did not undergo a repeated MRI scan, which may weaken the statistical power of a longitudinal study. More samples are warranted to offset this effect in future studies. Finally, our efforts were mainly concentrated on dFC. It is worth mentioning that emerging evidence demonstrates the co-evolutionary relationship between FC and local brain activity (23). Future studies could further explore co-evolutionary patterns between FC and local brain activity of LBNs enrolled in emotion regulation.

## Conclusions

Given the adaptive nature and network-level representation in the brain of emotion, exploring time-varying functional interactions is important to characterize emotion dysregulation in MDD. The present study identified several recurring functional interaction patterns among LBNs enrolled in emotion regulation and further investigated their abnormal temporal configuration as well as reconfiguration following antidepressant treatment in MDD. Furthermore, we found that predictive biomarkers of effective antidepressant treatment were embedded in the dynamic interactions among LBNs. These findings demonstrate that the dynamic interactions among LBNs serving for emotion regulation have the potential to be the target of clinical intervention for MDD.

## Data Availability Statement

The raw data supporting the conclusions of this article will be made available by the authors, without undue reservation.

## Ethics Statement

The studies involving human participants were reviewed and approved by the Local Institutional Review Boards (IRB) of Hangzhou Normal University. The patients/participants provided their written informed consent to participate in this study. Written informed consent was obtained from the individual(s) for the publication of any potentially identifiable images or data included in this article.

## Author Contributions

S-WX, LZ, DW, and ZT: concept and design. LZ, S-WX, and ZL: drafting of the manuscript. LZ, S-WX, ZL, and YX: critical revision of the manuscript for important intellectual content. LZ, CK, HLi, and CP: statistical analysis. DW, S-WX, and HLu: obtained the funding. LZ, XH, SF, and YW: administrative, technical, and material support. S-WX and DW: supervision. All authors: acquisition, analysis, and interpretation of data.

## Funding

This work was supported by the Zhejiang Medical and Health Science and Technology Project (2022KY1055), Natural Science Foundation of Zhejiang Province (LY17H180007), Key Medical Disciplines of Hangzhou (Clinical Cognitive Psychology), National Natural Science Foundation of China (81271503), Hangzhou Science and Technology Commission Foundation (20170533B06), and Affiliated Hospital of Hangzhou Normal University.

## Conflict of Interest

The authors declare that the research was conducted in the absence of any commercial or financial relationships that could be construed as a potential conflict of interest.

## Publisher's Note

All claims expressed in this article are solely those of the authors and do not necessarily represent those of their affiliated organizations, or those of the publisher, the editors and the reviewers. Any product that may be evaluated in this article, or claim that may be made by its manufacturer, is not guaranteed or endorsed by the publisher.
